# Spontaneous Cure of Cataract

**Published:** 1844-12-14

**Authors:** 


### SPONTANEOUS CURE OF CATARACT.

A stone-breaker had suffered from cataract from his youth. Whilst pursuing his occupation, he was struck by a splinter in the affected eye, and this gave rise to severe inflammation. He consulted a medical man, who, with a view of examining the eye, dropped into it a solution of belladonna. The pupil became largely dilated, aud at the same time the opaque lens fell into the anterior chamber, vision being immediately restored.—*Edinburgh Monthly Journal.*

